# Preparation of Cu/Cu_2_O/BC and Its Performance in Adsorption–Photocatalytic Degradation of Methyl Orange in Water

**DOI:** 10.3390/ma17174306

**Published:** 2024-08-30

**Authors:** Gang Du, Yarong Ding, Canhua Li, Lanyue Zhang, Jiamao Li, Minghui Li, Weichang Zhu, Chuan He

**Affiliations:** 1 College of Metallurgical Engineering, Anhui University of Technology, Maanshan 243032, China; antony618@163.com (G.D.); dingyarong12@163.com (Y.D.); zly921731@163.com (L.Z.); goodminghui@163.com (M.L.); 2 Anhui Key Laboratory of Low Carbon Metallurgy and Solid Waste Resource Utilization, Anhui University of Technology, Maanshan 243002, China; 3 College of Materials Science and Engineering, Anhui University of Technology, Maanshan 243002, China; lijiamao@ahut.edu.cn (J.L.); 13955569230@163.com (W.Z.); 4 Department of Mechanical and Electrical Engineering, Jiuquan Vocational and Technical College, Jiuquan 735000, China; hchuan@outlook.com

**Keywords:** Cu/Cu_2_O/BC composites, sodium hypodisulfite, adsorption–photocatalysis, methyl orange, wastewater treatment

## Abstract

In this study, we prepared a low-cost novel Cu/Cu_2_O/BC nanocomposite visible-light photocatalyst by the impregnation method using CuSO_4_·5H_2_O and rice husk biochar (BC) as raw materials and Na_2_S_2_O_4_ as a single reductant to improve the stability and dispersion of the Cu/Cu_2_O nanoparticles, in order to solve their aggregation tendency during photocatalysis. The morphology and structure of the Cu/Cu_2_O/BC were characterized using various analytical and spectroscopic techniques. The photocatalytic effect and cyclic stability of the synthesized photocatalyst on methyl orange (MO) removal were investigated under visible light radiation and various parameter conditions, including the mass ratio of BC to Cu/Cu_2_O, initial MO concentration, pH, temperature, and catalyst dosage. The results show that the synthesized Cu/Cu_2_O/BC nanocomposite composed of Cu/Cu_2_O spherical particles was loaded on the BC carrier, which has better stability and dispersion. The best adsorption–photocatalytic effect of the Cu/Cu_2_O/BC is exhibited when the mass ratio of BC to Cu/Cu_2_O is 0.2. A total of 100 mg of Cu/Cu_2_O/BC can remove 95% of the MO and 88.26% of the COD in the aqueous solution at pH = 6, T = 25 °C, and an initial MO concentration of 100 mg/L. After five cycles of degradation, the MO degradation rate in the sample can still remain at 78.41%. Both the quasi-secondary kinetic model and the Langmuir isothermal adsorption model describe the adsorption process. Additionally, the thermodynamic analysis demonstrates that the photocatalytic process follows the quasi-primary kinetic model and that the removal process is of spontaneous heat absorption. The photocatalyst described in this paper offers a cost-effective, easily prepared, and visible-light-responsive solution for water pollution treatment.

## 1. Introduction

Particular kinds of commonly found water pollutants are organic dyes, which are emitted by the food-processing, ink-printing, textile-dyeing, and make-up industries [1,2]. Azo dyes, which are included among these organic dyes, are both toxic and carcinogenic, especially methyl orange (MO), which is considered a dangerous aromatic compound with the potential to induce various diseases, such as hypertension and tumors, in humans. Meanwhile, organic azo dyes block sunlight from penetrating through the water surface, affecting the survival of aquatic plants and animals. It is estimated that due to rapid industrialization and urbanization, more than 100,000 dyestuffs are currently marketed, and at least 700,000 tonnes of each type of dyestuff are produced annually [3]. More than 60% of dyestuffs are used by textile and dyeing processors, with approximately 20% released into water bodies as effluents throughout the manufacturing process. Practical solutions to remove organic pollutants from water, especially waste dyes, are therefore urgently needed.

Photocatalysts can produce highly oxidizing substances in water under sunlight and mineralize organic pollutants through oxidation, which is one of the most thorough and economical water purification methods used in the industries of renewable resources and semiconductor materials. Typical p-type semiconductors, such as Cu_2_O, have a small bandgap of about 2.0–2.2 eV [4], which makes them suitable for use as visible light photocatalysts because of their strong light absorption and excellent tunability in the visible light region. At present, the main synthesis methods for Cu_2_O include the liquid-phase reduction method [5], the electrochemical method [6], the hydrothermal method [7], and the electrodeposition method [8]. Because of its ease of use, the liquid-phase reduction approach is the most popular among these methods. The main reductants often used in the liquid-phase reduction method are NaBH_4_ [9,10], N_2_H_4_ [11], Vitamin C (Vc) [12], etc. However, NaBH_4_ is expensive and unfavorable for industrial promotion. N_2_H_4_, although inexpensive, is itself a highly toxic substance to living organisms. Furthermore, Vc is not only expensive, but it is also consumed in large quantities. These limitations have undoubtedly hindered the large-scale preparation and application of Cu_2_O as a photocatalyst. At the same time, it has been reported that the redox potential of Na_2_S_2_O_4_ can be as low as −1.12 V, with strong reducibility [13] under certain conditions, and since its price is only one-tenth of that of NaBH_4_ [14], the Na_2_S_2_O_4_ reductant can be utilized as an environmentally friendly and low-cost alternative to the other reductants mentioned above [15,16]. The production of Cu_2_O by the use of Na_2_S_2_O_4_ as a reductant has not yet been the subject of research.

However, because of Cu_2_O’s low forbidden band width, which limits its applicability in photocatalysis, its particles tend to aggregate throughout the process and the photogenerated electrons/holes are prone to complexation. To sustain Cu/Cu_2_O particles steadily, it is crucial to use an appropriate carrier. The practical application of this approach is limited by its high cost, although graphene oxide is a carbon carrier with remarkable chemical stability. In contrast, ricehull charcoal is less expensive and more environmentally friendly, and it possesses a large specific surface area, high porosity, many surface-oxygenated groups, and stable chemistry. These characteristics make it an inexpensive, easily obtained, high-performing adsorbent material that is more frequently used in the wastewater treatment industry.

Based on this, in this study, the Cu/Cu_2_O/BC nanocomposites were synthesized by the impregnation method, with Na_2_S_2_O_4_ used as a single reducing agent. XRD, XPS, SEM, BET, FTIR, UV-Vis, and other techniques were used for the description of the structure and composition of the Cu/Cu_2_O/BC materials produced. The effects on the adsorption–photocatalysis of Cu/Cu_2_O/BC on the MO removal by different mass ratios of BC to Cu/Cu_2_O, the initial concentration, the pH, and the temperature were investigated by single-factor experiments. The kinetics, adsorption isotherm, and thermodynamics were analyzed. The chemical and physical properties of the reactants, determined by FTIR, XRD, and FESEM-EDS, were used to study the potential reaction mechanism.

## 2. Materials and Methods

### 2.1. Raw Materials

The copper sulfate pentahydrate (CuSO_4_·5H_2_O, GR) and sodium dithionite (Na_2_S_2_O_4_, AR) used in this study are produced by Shanghai McLean Biochemical Science and Technology Co., Ltd. (Shanghai, China) Rice husk biochar (BC) is produced by Jieshou Agile Environmental Protection Science and Technology Co. Ltd. (Jieshou, China), and methyl orange (CHN_3_SO_3_Na, AR) and sodium hydroxide (NaOH, AR) are produced by Sinopharm Chemical Reagent Co. Water (Shanghai, China) that was deionized was utilized in the experiment.

### 2.2. Preparation of Cu/Cu_2_O/BC

Using 15.75 g CuSO_4_-5H_2_O in 100 mL deionized water, an aqueous solution containing 4 g/L of Cu(II) is prepared. In 20 mL of deionized water, an additional 1.2 g of NaOH and 1.2 g of Na_2_S_2_O_4_ are weighed and thoroughly dissolved with rapid stirring. The biochar is sieved through a 200-mesh sieve, and a determined amount of 0.04, 0.08, 0.12, 0.16, 0.2, and 0.4 g of biochar is poured into a solution of 100 mL of Cu(II) and impregnated for 6 h. Stirring is required at intervals during the process of the impregnation and adsorption to prevent uneven adsorption on the bottom of the sink. After the impregnation is completed, the solution is heated to 50 °C in nitrogen, and then the NaOH/Na_2_S_2_O_4_ mixed solution is slowly dripped into the solution and stirred at 400 r/min for 10 min. After removing the reaction products and rinsing them three times in deionized water and anhydrous ethanol to make them neutral, they are placed in the vacuum drying oven set at 60 °C for 6 h.

The Cu/Cu_2_O/BC nanoparticles are then obtained by grinding. To examine the impact of mass ratio of BC to Cu/Cu_2_O on MO removal, six Cu/Cu_2_O/BC composites with mass ratios of BC to Cu/Cu_2_O of 0.1, 0.2, 0.3, 0.4, 0.5, and 1.0 are prepared, termed as Cu/Cu_2_O/C-*x*, where *x* is the mass ratio of BC to Cu/Cu_2_O.

### 2.3. Adsorption and Photocatalysis Experiments

The adsorption and photocatalysis of Cu/Cu_2_O/BC to MO were investigated in the presence of visible light, with wavelengths ranging from 400 to 780 nm. In order to examine the impacts of various parameters on the adsorption–photocatalytic efficacy of MO removal, the adsorption experiments were also conducted in a dark environment. The parameters included adsorbent dose, starting concentration, pH value, temperature, and contact duration.

In the adsorption experiments, 0.1 mol/L HCl and 0.1 mol/L NaOH are utilized to modify the pH of 100 mL of simulated wastewater that is added to a quartz cup at a specific concentration. A certain amount of Cu/Cu_2_O/BC is weighed and added into the solution, which is stirred using a magnetic stirrer set at 150 r/min, and the reaction is conducted in an air environment at a constant temperature. After a specified time, 3 mL of the sample is taken with a pipette gun and passed through a 0.45 µm needle filter to remove particles, and the absorbance is measured at 462 nm with a visible spectrophotometer.

In the photocatalytic experiments, 100 mL of MO solution and a certain amount of catalyst are added into a 100 mL quartz cup, which is first stirred at 150 r/min for 60 min in a dark environment to reach the equilibrium of adsorption and desorption, and then the photocatalytic degradation reaction is carried out at 25 °C under visible light radiation (LED lamp, 45 W). After a certain interval, 3 mL of sample is taken out using a pipette gun and filtered using a 0.45 μm needle filter to remove particles, and the resulting solution is used for the determination of the photocatalytic analysis. All the experiments involved in this study were repeated three times and the mean values were recorded.

Equation (1) is used to calculate the MO removal rate (removal efficiency), as follows:

(1) $$RE\%=\frac{C_{0}-C_{t}}{C_{0}}\times 100\%$$

where the MO concentrations before and at reaction times *t* (min or h) are denoted by the numbers *C*_0_ (mg/L) and *C_t_* (mg/L).

The removal capacity of the material for MO is calculated according to Equation (2), as follows:

(2) $$q_{t}=\frac{C_{0}-C_{t}}{m}\times V$$

where *q_t_* (mg/g) is the amount of MO adsorbed at time *t*, *m* (g) is the mass of the adsorbent, and *V* (L) is the volume of the methyl orange solution.

**Figure 1:**
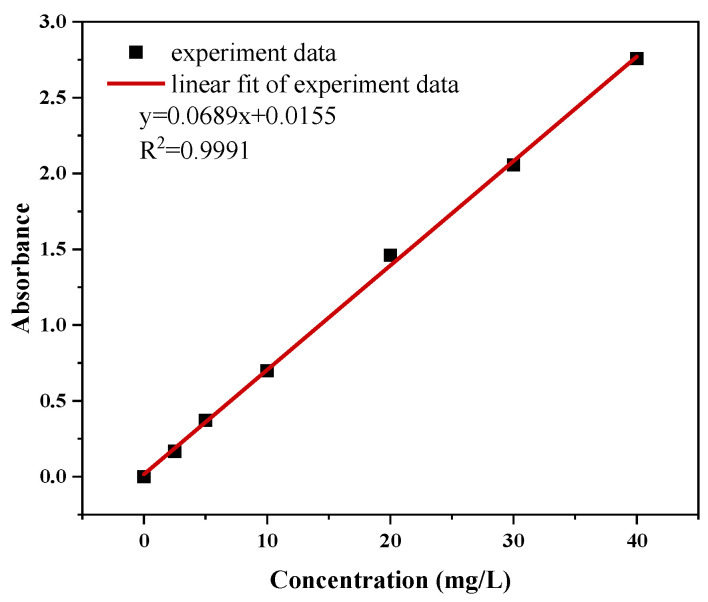
Standard absorbance curve of MO removal at 462 nm.

The standard curve determination includes determining the concentration of 0, 2.5, 5, 10, 20, 30, and 40 mg/L MO solution, measuring the absorbance, plotting the calibration curve of concentration relative to absorbance, as shown in Figure 1, and establishing a linear regression equation.

### 2.4. Test and Characterization

The morphology of Cu/Cu_2_O/BC is characterized using SEM (MIRA3, TESCAN, Brno, Czech Republic) and TEM (Talos F200X, FEI, Lausanne, Switzerland). The crystal composition in the material can be determined by XRD (6000, Shimadzu, Kyoto, Japan). The XPS (Thermo Scientific K-alpha, Waltham, MA, USA) is the study of changes in chemical valence electrons on the material surface. The specific surface area of the adsorption data points is calculated using BET. FTIR (Nicolet iS20, ThermoFischer, Waltham, MA, USA) is used to qualitatively detect functional groups on the material surface, and the light absorption properties of the products are determined using a UV-Vis DRS (UV-3600, Shimadzu, Kyoto, Japan). The zeta potential of the materials at different pH values is determined using the solid-state addition method (Zetasizer Nano ZS90, Malvern, Enigma Business Park, UK).

## 3. Results and Discussion

### 3.1. Material Characterization

#### 3.1.1. XRD and Raman Analyses

**Figure 2:**
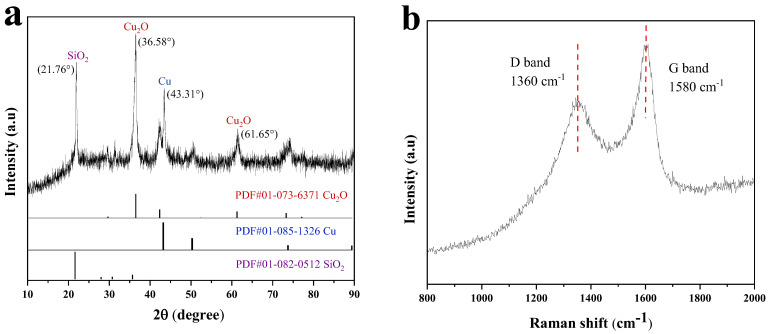
XRD patterns (**a**) and Raman spectrum (**b**) of Cu/Cu_2_O/BC.

Figure 2a shows the XRD spectra of the Cu/Cu_2_O/BC composites. The diffracted characteristic peaks located at 2*θ* = 29.65°, 36.58°, 42.49°, and 61.65° correspond to the (110), (111), (200), and (220) lattice planes of the Cu_2_O crystalline phase (PDF#01-073-6371) of the sample, respectively, and the diffracted characteristic peaks located at 2*θ* = 43.31° and 50.37° correspond to the Cu (111) and (200) lattice planes of the crystalline phase (PDF#01-085-1326) of the sample [17], respectively. It is also observable that the diffraction peaks of the SiO_2_ phase (PDF#01-082-0512) are located at 21.76°, 28.21°, 30.96°, and 35.91, respectively, where SiO_2_ is the substance present in the rice husk biochar. The sample’s Raman spectrum is displayed in Figure 2b. The two prominent peaks, which are centered at 1360 and 1580 cm^−1^, respectively, correspond to the distinctive signals of the disordered (D-band) and graphite (G-band) bands of carbon [18]. This means that the Raman spectra provide direct evidence that carbon materials are present. Based on the XRD and Raman spectrograms, it is clear that the prepared samples are composites of Cu, Cu_2_O, and biochar, referred to as Cu/Cu_2_O/BC.

#### 3.1.2. XPS Analysis

**Figure 3:**
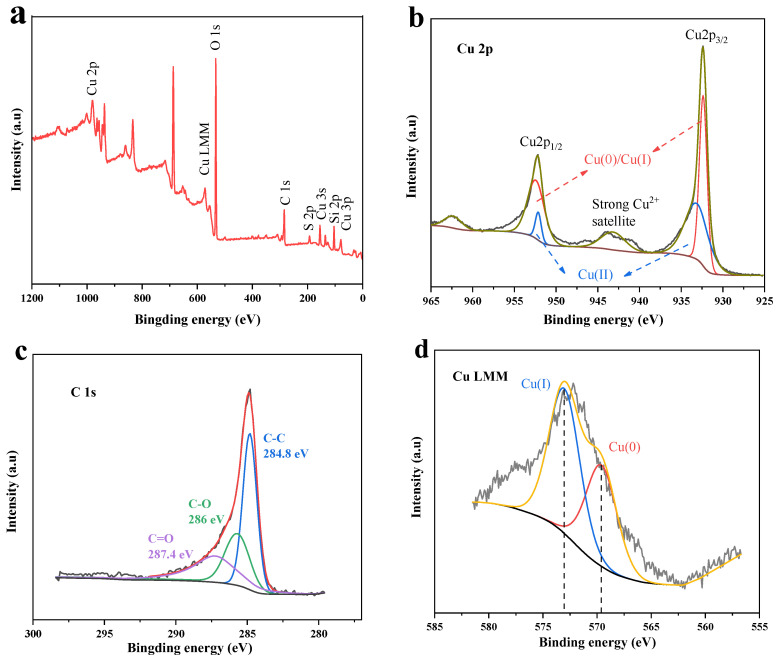
XPS patterns of Cu/Cu_2_O/BC: (**a**) XPS full spectrum, (**b**) Cu 2p, (**c**) C 1s, and (**d**) Cu LMM.

An XPS analysis was used to make a further assessment of the elemental composition and the state of chemical bonding of the Cu/Cu_2_O/BC nanoparticles. The findings are displayed in Figure 3. The elements Cu, O, C, and Si were present in the Cu/Cu_2_O/BC sample, as shown in Figure 3a, which is in line with the findings of the XRD test. In particular, Si was a component of the biochar, and the characteristic peak intensity of the elemental C in Cu/Cu_2_O/BC was obviously higher than that of the Cu/Cu_2_O, indicating that the elemental C in this sample mainly originated from the carrier biochar.

The high-resolution XPS spectra of the Cu/Cu_2_O/BC in the Cu 2p and C 1s areas, respectively, are shown in Figure 3b,c. Observed at 931.25 eV and 951 eV, respectively, the Cu 2p peaks with pronounced spin-orbit splitting (Δ = 19.75 eV) correspond to Cu 2p_3/2_ and Cu 2p_1/2_, with photoelectron compositions from Cu(0) and Cu(I). This suggests that the Cu(0)/Cu(I) splitting at 19.75 eV is close to the standard separation of 19.8 eV [19,20]. The Cu(II) satellite peak is observable at 943 eV. Since the surface of the sample was usually exposed to air, it can be inferred that the surface oxidation of the sample produced Cu(II). The C 1s spectra’s three peaks at 284.8, 286.0, and 287.7 eV are ascribed to C–C, C–O, and C=O, respectively [21,22].

On the basis of the Cu 2p spectra alone, it was challenging to identify the presence of Cu(0) or Cu(I), since Cu(0) and Cu(I) have similar binding energies at 932.5 eV [23]. Furthermore, the Rochelle electron spectra were used to ascertain the various chemical states of Cu in order to investigate the chemical grouping of the samples in more detail. Cu(0) and Cu(I) are represented by the peaks at 568.5 and 572.3 eV, as shown in the Cu LMM valence electronic spectrum in Figure 3d [24,25].

#### 3.1.3. SEM-EDS Analysis

**Figure 4:**
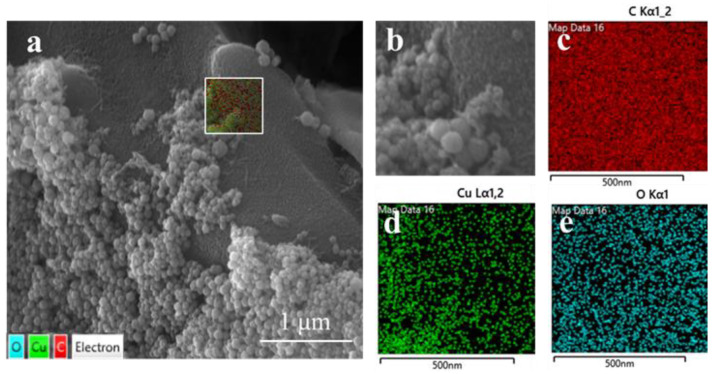
SEM images of the synthesized Cu/Cu_2_O/BC (**a**,**b**), and element mapping of C (**c**), Cu (**d**), and O (**e**).

Figure 4 displays the Cu/Cu_2_O/BC nanocomposite’s SEM picture. The SEM image of Cu/Cu_2_O/BC is displayed in Figure 4a, and the further enlarged image of the chosen location in Figure 4a is displayed in Figure 4b. It can be observed that many spherical particles are piled up on the irregular plate, the surface of the plate is rough, and the size of the spherical particles is about 100 nm. The material can be characterized by the EDS pattern, as shown in Figure 4b–e. The EDS pattern shows that the sample consists of the elements Cu, O, and C, with C displaying the highest content and a uniform distribution. It was determined that this plate was a biochar, and that the element of Cu was mainly distributed on the spherical particles. The spherical particles were judged to be Cu and cuprous oxide.

#### 3.1.4. BET Analysis

**Figure 5:**
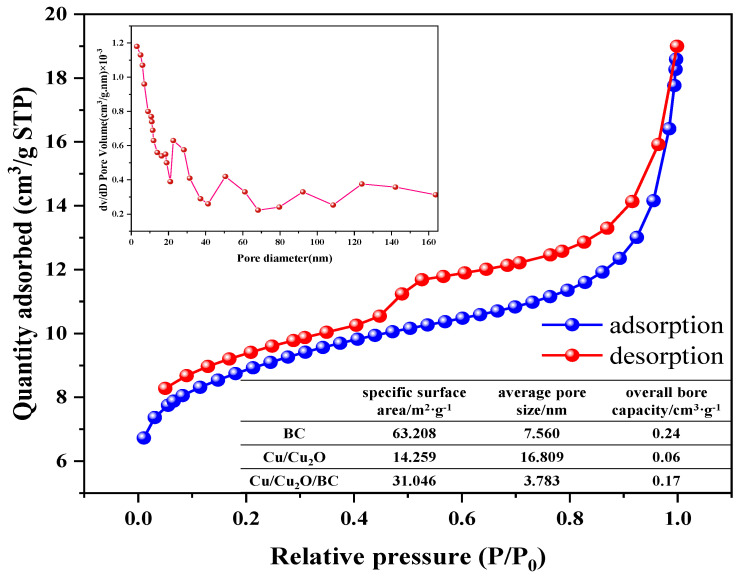
N_2_ adsorption–desorption isotherms of Cu_2_O and Cu/Cu_2_O/BC.

The N_2_ adsorption–desorption isotherm was used for the investigation of the specific surface area and pore size distribution of the samples. The result for Cu/Cu_2_O/BC is shown in Figure 5, which exhibits a type IV isotherm with a bulge after one section of the curve and a hysteresis loop in the middle, corresponding to the system of capillary coalescence of porous adsorbents. According to the IUPAC classification [26], the hysteresis ring belongs to H-4 porous solids, which do not have an obvious saturated adsorption platform, and whose pore structure is irregular [1]. The specific surface area of the Cu/Cu_2_O/BC sample is 31.046 m^2^/g, and its average pore size is 3.783 nm, according to the BET model. Its large specific surface area provides abundant active sites conducive to adsorption–photocatalytic reactions, which contributes to enhancing the adsorption and photocatalytic performance.

#### 3.1.5. FTIR Analysis

**Figure 6:**
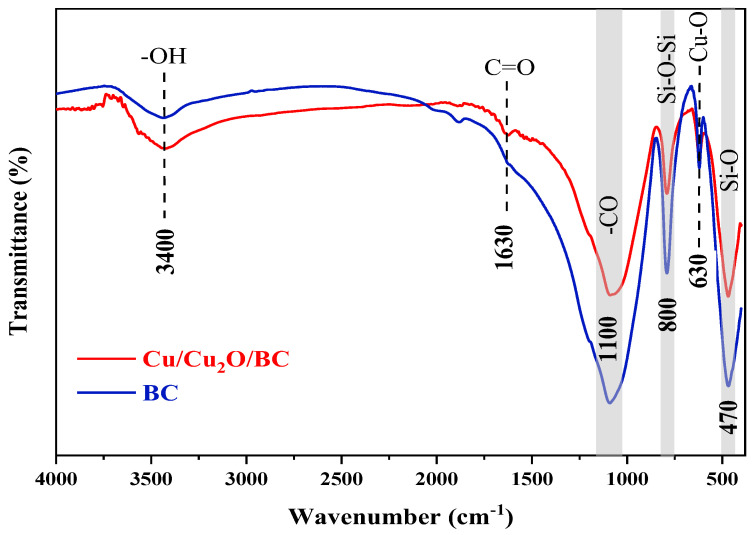
FTIR of Cu/Cu_2_O/BC and BC.

The functional groups of Cu/Cu_2_O/BC were determined by the FTIR spectra, as shown in Figure 6, and the red and blue lines are the FTIR spectra of Cu/Cu_2_O/BC and BC, respectively. The carboxyl group or C=O stretching vibration of ketones, aldehydes, and lactones was attributed to the characteristic peak of the stretching vibration of the surface hydroxyl group (–OH) at 3400 cm^−1^ [27] and absorption peaks at 1630 cm^−1^. These findings suggest that the surface of the rice husk BC contained a large number of oxygenated functional groups, such as –OH, –COOH, and –COH, as well as other oxygen-containing functional groups [28]. The peaks of the BC are distributed at 1100, 800, and 470 cm^−1^, among which 1100 cm^−1^ corresponds to the stretching vibration of –CO, perhaps caused by the lignin ether bond O–C–O in the rice husk biochar [29]. The Si–O–Si bond exhibits a peak at 800 cm^−1^ during its symmetric stretching vibration [30], while the symmetric stretching vibration of the Si–O bond displays a peak at 470 cm^−1^.

The presence of Cu_2_O was demonstrated by the Cu/Cu_2_O/BC spectra at 630 cm^−1^, which matches the Cu–O stretching vibration peak [31]. In addition, the absorption peaks of the –OH, –COOH, –COH, and –CO functional groups in the biochar at 1100, 800, and 470 cm^−1^ in the FTIR spectrum of the Cu/Cu_2_O/BC were observed. These oxygen-containing functional groups provide adsorption sites for dyes in the environment.

#### 3.1.6. UV-Vis DRS Analysis

**Figure 7:**
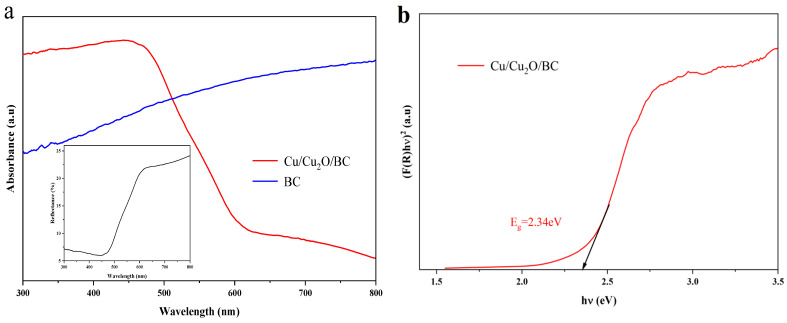
(**a**) Uv-vis DRS of BC and Cu/Cu_2_O/BC, inset: diffuse reflectance of Cu/Cu_2_O/BC; (**b**) Tauc plot of Cu/Cu_2_O/BC.

The material’s light absorption characteristics are reflected in the UV-Vis DRS spectra, and the wider the range of the absorption wavelengths, the higher the utilization of light. The absorption spectral curves of the BC and Cu/Cu_2_O/BC corresponding to the forbidden bandwidths are shown in Figure 7, where it can be observed that the light absorption range of the BC is wide, while the light absorption of the Cu/Cu_2_O/BC sample begins to decline at 450 nm and reaches its lowest point when the absorption edge extends to 600 nm. Therefore, the light absorption efficiency of the Cu/Cu_2_O/BC composite is higher than that of the BC. The absorbance was obtained by transforming the diffuse reflectance of the solid powder of the Cu/Cu_2_O/BC (with the inset given in Figure 7a) using the Kubelka–Munk (K-M) reflection theory [32,33]. The band gap values of the Cu/Cu_2_O/BC composites were calculated using the Tauc function, as shown in Equations (3) and (4) [34,35]:

(3) $$F(R)=\frac{{\left(1-R\right)}^{2}}{2R}$$

(4) $${\left[F(R)hv\right]}^{1/n}=A(hv-E_{g})$$

where F(R) is the Kubelka–Munk function, R is the reflection coefficient, *hv* is the photon energy, A is a constant, *E_g_* is the optical bandgap, *n* = 2 represents indirect leaps, and *n* = 1/2 represents direct leaps [36]. Studies report that Cu_2_O exhibits a directly permissive transition band type [14,37]. Therefore, the bandgap value of the synthesized particles is determined by (F(R)*hν*)^2^ with respect to *hν*. Extrapolating the straight line permitting the direct leaps partially extrapolated to the x-axis, the calculated band gap value is 2.34 eV with an absorption edge of 620 nm, according to the equation *E_g_* = 1240/λ, as shown in Figure 7b.

### 3.2. Adsorption and Photocatalysis

**Figure 8:**
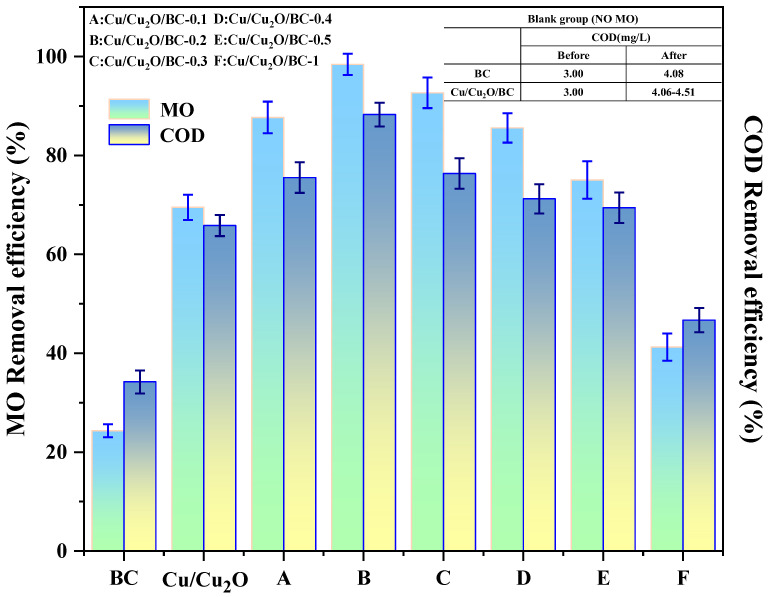
Effects of different mass ratios of BC to Cu/Cu_2_O on the removal rate of MO and COD.

Six composites with various mass ratios were made in order to examine the impact of the mass ratio of BC to Cu/Cu_2_O on the MO removal. The MO and COD removal outcomes of these composites under visible light are displayed in Figure 8. The values for the removal of MO by the BC or the Cu/Cu_2_O alone were 21.34% and 69.52%, respectively, indicating that Cu/Cu_2_O is more favorable for the photocatalytic degradation of MO than BC. The outcomes of the MO removal process using the Cu/Cu_2_O/BC composite demonstrate that the removal of MO is positively impacted by a certain amount of biochar loading. When the mass ratio of BC to Cu/Cu_2_O is 0.2 and the reaction is equilibrated, the highest MO removal rate of 95% is achieved. However, when the mass ratio of BC to Cu/Cu_2_O increases to 0.3, the removal rate begins to show a decreasing trend. This is because the excess biochar increases the solution turbidity and decreases the light transmission of the reaction system, which thus lessens the catalyst’s ability to degrade MO. A blank group without MO was also set up to observe the stability of the BC and the Cu/CU_2_O/BC, which maintained relatively reliable stability during the photocatalytic reaction, as shown by the comparison of the before and after results for chemical oxygen demand (COD). Furthermore, the COD removal rate showed an increasing and then a decreasing trend with the increase in the mass ratio of BC to Cu/Cu_2_O, which was consistent with the photodegradation pattern of MO. In addition, the best COD removal rate, of 88.26%, was achieved by the Cu/Cu_2_O/BC-0.2 for the MO, in which the initial concentration of COD of MO was 113 mg/L, and after the reaction, the concentration of COD was reduced to 13.24 mg/L, which meets the sewage discharge standard. Therefore, the optimized mass ratio of BC to Cu/Cu_2_O in the Cu/Cu_2_O/BC was 0.2, which had the best degradation effect and was subsequently selected for the adsorption and photocatalysis experiments.

#### 3.2.1. Dark Adsorption and Kinetics

The mass ratio of 0.2 of BC to Cu/Cu_2_O of Cu/Cu_2_O/BC was used as the adsorbent to simulate dye wastewater with a certain MO configuration to study the effects of the time required for adsorption, the initial dye concentration of MO, the reaction temperature, and the pH of the solution on the MO adsorption process.

**Figure 9:**
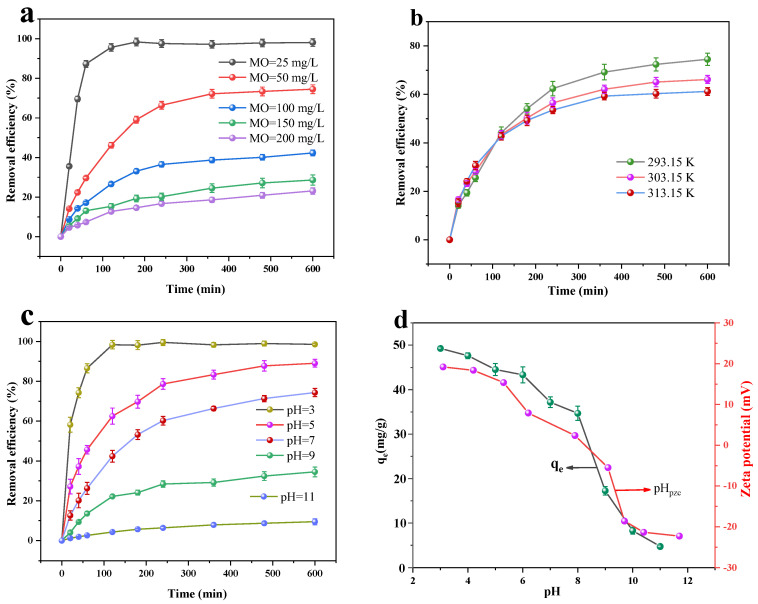
Effects of initial MO concentration (**a**), temperature (**b**), and pH (**c**) on adsorption efficiency and zeta potential (**d**).

The MO dark adsorption experiments were used to investigate the impact of the starting dye concentration on the MO’s adsorption by the Cu/Cu_2_O/BC. The experiments were conducted at pH = 6, at T = 25 °C, and with 100 mg of Cu/Cu_2_O/BC dosing. Because of the limited number of adsorptive sites on the surface of the adsorbent and the resulting prolonged equilibration time, as shown in Figure 9a, the MO removal rate fell as the MO concentration rose. The MO solution was first treated by Cu/Cu_2_O/BC, and there were many accessible contact sites on the surface, indicating a significant MO adsorption capacity. The MO dye molecules rapidly attached to the surface of Cu/Cu_2_O/BC. The adsorption response approached equilibrium in 120 min at a starting MO concentration of 25 mg/L, at which point the MO removal rate reached 95.67%. However, the MO removal rate dropped to 23.10% when the MO was treated at the initial concentration of 200 mg/L. During the adsorption process, the active sites on the surface of the adsorbent were rapidly blocked, reducing the adsorption driving force, thus hindering the continuous adsorption of the remaining MO dye ions.

The findings are displayed in Figure 9b. The effects of the ambient temperatures of 293.15, 303.15, and 313.15 K on the MO adsorption process were examined for the initial conditions of pH = 6, MO initial concentration of 50 mg/L, and Cu/Cu_2_O/BC dosage of 100 mg, respectively. It is clear that during the initial phase of the adsorption process, the rate of adsorption rises with the temperature. As the adsorption–desorption reaction continues, the efficiency of the removal of the MO by the Cu/Cu_2_O/BC gradually decreases from 74.5% to 61.21% with the rise in temperature. This decrease is due to the self-enhancement of the desorption during the adsorption process, suggesting that the reaction is exothermic [38]. In addition, the adsorption between the Cu/Cu_2_O/BC active sites and the MO molecules reduces as temperature rises [39,40], suggesting that this kind of adsorption is reversible. In this case, the temperature dependence of the adsorption is weaker than that of the desorption.

When the initial concentration of MO was 50 mg/L and the temperature was 25 °C, the effects of different pH values on the adsorption of MO were investigated using 100 mg of Cu/Cu_2_O/BC. The results are displayed in Figure 9c,d. It is evident that the adsorption effect of the Cu/Cu_2_O/BC on the MO is enhanced with the decrease in the pH value. The maximum MO removal rate reaches 98.56% when the pH is 3, and the maximum adsorption *q*_max_ is 49.28 mg/g. This may be compared with the studies by Khan et al. [41] and Zhu et al. [42], in which the adsorption values of 28.2 mg/g and 29.5 mg/g increased the MO removal rate by 74.75% and 67.06%, respectively. The elimination of MO reduces quickly, to 34.52%, when the pH of the solution is raised to 9. The reason for this is that when the pH value exceeds 7, the higher concentration of OH^-^ ions in the solution competes with the anionic dye molecules for available active sites on the adsorbent surface, leading to a reduction in the amount of dye molecules that can adsorb. Adsorbents with positively charged surfaces (pH < pH_pzc_) are more efficient in removing MO than those with negatively charged surfaces. The high rate of removal of MO by the Cu/Cu_2_O/BC at pH of 3–7 also confirms the pH_pzc_ concept that the adsorption of anionic dyes, such as MO, is usually favored in the range of positively charged surfaces (pH = 0–7), whereas the competition of anionic MO dyes with the OH^-^ group at pH > pH_pzc_ hinders the adsorption efficiency. Thus, the adsorption process is carried out at favored pH < pH_pzc_ conditions [43].

**Figure 10:**
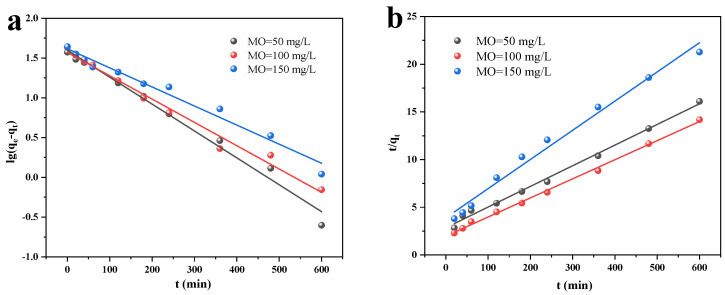
Linear fitting of adsorption kinetics: (**a**) quasi-first-order kinetics, and (**b**) quasi-second-order kinetics.

The kinetics of MO desorption by Cu/Cu_2_O/BC were investigated by using the adsorption results with time, according to the proposed primary and secondary kinetic models. The adsorption kinetics’ linear fitting is displayed in Figure 10, and the primary parameters of the kinetic model fitting are displayed in Table 1. The proposed primary kinetic model’s linear forms and secondary kinetic model are presented in Equations (5) and (6), as follows:

(5) $$\ln(q_{e}-q_{t})=\ln q_{e}-k_{1}t$$

(6) $$\frac{t}{q_{t}}=\frac{1}{k_{2}q_{e}^{2}}+\frac{t}{q_{e}}$$

where *q_e_* (mg/g) is the adsorption capacity at equilibrium, *q_t_* (mg/g) is the adsorption capacity recorded at a given time *t* (min), and *k*_1_ (1/min) and *k*_2_ (mg/g·min) are the rate constants for the proposed first- and second-order kinetic equations.

According to the data in Table 1, both the proposed first-order and second-order kinetic equations exhibit correlation coefficients (R^2^) exceeding 0.95. This suggests that both kinetics are partly capable of describing the adsorption process, and that the adsorption behaviors may be related to both chemical and physical adsorptions. However, the correlation coefficients of the proposed second-order kinetic model (R^2^ > 0.99) are even closer to 1 when compared to the values obtained from the fitted calculation of qm. This indicates that the proposed second-order kinetic model is more suitable for the adsorption of MO by Cu/Cu_2_O/BC.

**Figure 11:**
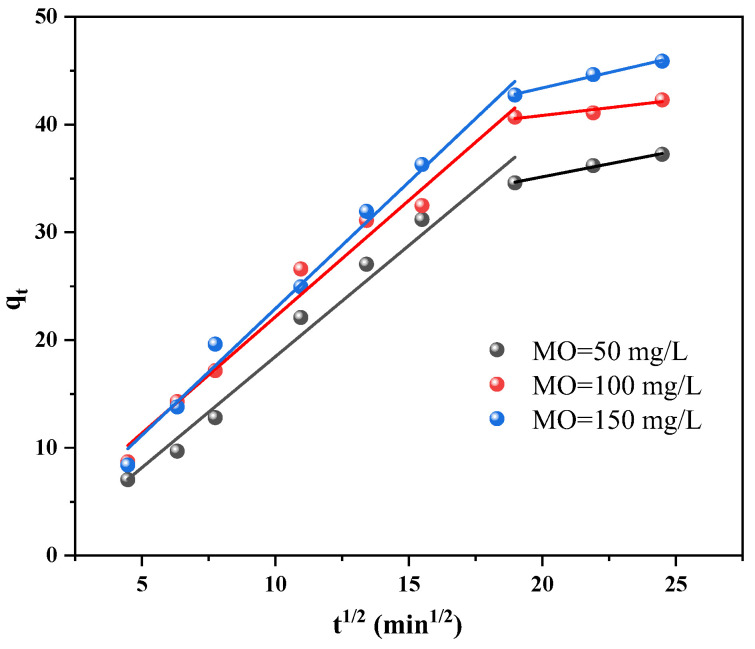
Internal diffusion model.

Figure 11 shows the results of the fitting of the internal diffusion model for the Cu/Cu_2_O/BC particles, which can indicate the rate-limiting step and diffusion mechanism of the adsorbent. The relevant parameters of the internal diffusion model for Cu/Cu_2_O/BC are shown in Table 2. In which, I denotes a constant proportional to the adsorbent’s boundary layer thickness, and all the values of I in the table are non-zero, indicating that in the process of the adsorption of MO by Cu/Cu_2_O/BC, internal diffusion is not the only way to limit the reaction rate. There are two stages to this material’s adsorption process: liquid film diffusion and internal diffusion. The higher adsorption rate in the first phase is due to the fact that MO is adsorbed on the surface of Cu/Cu_2_O/BC through diffusion within the boundary layer. The similarity between the slopes of the three sets of fitted curves for the first phase of MO with different initial concentrations suggests that the initial concentration does not impact the adsorption rate during this phase. In the second stage, the adsorption rate is primarily controlled by intra-particle diffusion. With the molecules diffusing into the interior of the catalyst, the active sites are gradually saturated, and the adsorption rate is obviously reduced. Furthermore, the rate of diffusion within the particles is influenced by the MO concentration, and the Cu/Cu_2_O/BC binding sites are gradually saturated near equilibrium.

#### 3.2.2. Adsorption Isotherms and Thermodynamic Analyses

The effects of the adsorption behavior of the Cu/Cu_2_O/BC on the MO were analyzed using the Langmuir and Freundlich models [44]. Equations (7) and (8) depict the linear forms of the Langmuir and Freundlich models, respectively.

(7) $$\frac{C_{e}}{q_{e}}=\frac{1}{q_{\max}k_{L}}+\frac{C_{e}}{q_{\max}}$$

(8) $$\ln q_{e}=\ln k_{F}+\frac{1}{n}\ln C_{e}$$

Langmuir’s dimensionless constant *R_L_* is calculated using Equation (9),

(9) $$R_{L}=\frac{1}{1+k_{L}C_{e}}$$

where the MO concentration at equilibrium is *C_e_* (mg/L), the adsorption capacity at equilibrium is *q_e_* (mg/g), the maximum adsorption capacity at adsorption saturation is *q*_max_ (mg/g), and the Langmuir and Freundlich adsorption equilibrium constants are *k_L_* (1/min) and *k_F_* (mg/g·min).

**Figure 12:**
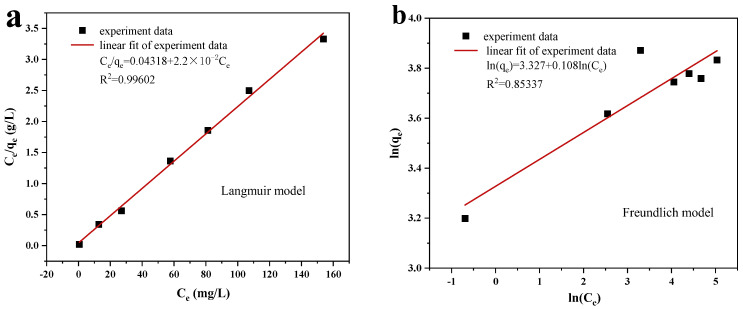
Equilibrium modelling of MO removal by Cu/Cu_2_O/BC: (**a**) Langmuir model, (**b**) Freundlich model.

Figure 12 illustrates the linear fitting for the Langmuir and Freundlich models, with the associated fitting parameters summarized in Table 3. According to the data from Figure 11 and Table 3, the Langmuir model exhibits a superior correlation coefficient (R^2^), suggesting that the adsorption of Cu/Cu_2_O/BC onto MO aligns more closely with the Langmuir model, which implies a mechanism of chemical adsorption. This suggests that the adsorption sites on the surface are uniform and that there is an absence of interaction among the adsorbates. Additionally, the adsorption process was deemed favorable, as indicated by the *R*_L_ value, which ranged between 0 and 1. Utilizing the Langmuir model, the maximum adsorption capacity of the MO (*q*_max_) was calculated to be 45.454 mg/g.

To determine whether an adsorption process is feasible and spontaneous, thermodynamic studies are necessary. Equation (10) through (12) may be used to compute the thermodynamic parameters, which include the Gibbs free energy (Δ*G*^0^), enthalpy (Δ*H*^0^), and entropy change (Δ*S*^0^), as follows:

(10) $$\Delta G^{0}=-RT\;\ln_{k}$$

(11) $$K_{c}=\frac{q_{e}}{C_{e}}$$

(12) $$\ln K_{c}=\frac{\Delta S^{0}}{R}-\frac{\Delta H^{0}}{RT}$$

where *R* (8.314 J/mol·K) is the universal gas constant, *T* (K) is the solution temperature, and $K_{c}\;\left(L/g\right)$ is the thermodynamic equilibrium constant.

**Figure 13:**
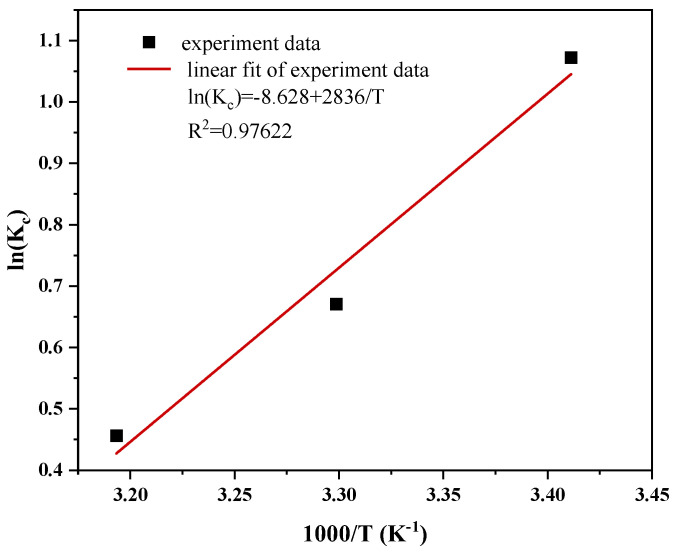
Van’t Hoff plot of Cu/Cu_2_O/BC for removing MO.

Figure 13 depicts the van’t Hoff diagram for 1000/T. Table 4 shows the thermodynamic parameters Δ*H*^0^ and Δ*S*^0^, which are computed based on the slope and intercept. The basically exothermic nature of the adsorption process is confirmed by the negative value of Δ*H*^0^ (−23.578 kJ/mol). Additionally, as the MO molecules adsorb on the Cu/Cu_2_O/BC surface, the adsorption system’s stoichiometry decreases, which accounts for the negative value of Δ*S*^0^ (−71.733 J/mol·K). Moreover, the spontaneous and thermodynamically advantageous adsorption of MO on Cu/Cu_2_O/BC is shown by the negative value of Δ*G*^0^ in the temperature range of 293.15–313.15 K. The fact that the adsorption occurs spontaneously when the temperature is raised from 293.15 K to 313.15 K and the Δ*G*^0^ increases from −9.831 to −2.889 kJ/mol confirms the adsorption [43]. The data also show that *|*Δ*H*^0^*|<|T∙*Δ*S*^0^|, indicating that entropy, as opposed to enthalpy, is primarily responsible for controlling the adsorption process.

#### 3.2.3. Photocatalysis and Kinetics

**Figure 14:**
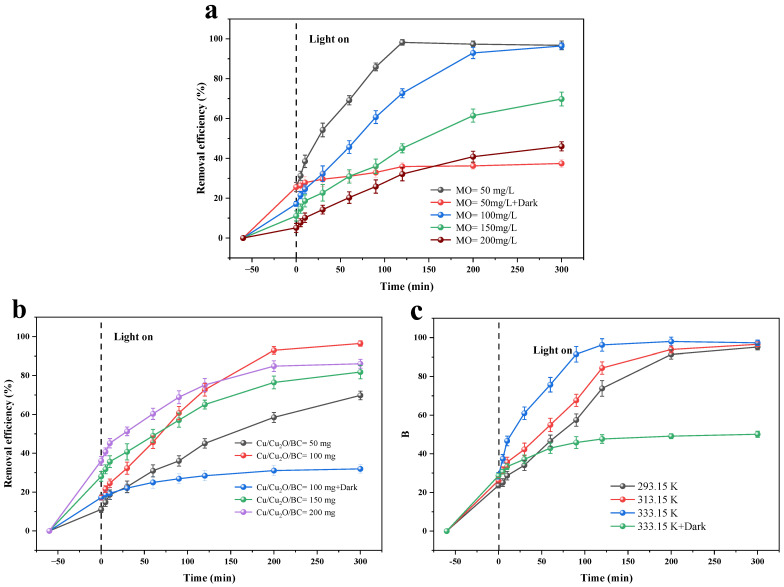
Effects on photocatalytic efficiency of Cu/Cu_2_O/BC by: (**a**) initial concentration, (**b**) dosage, and (**c**) temperature.

Batch removal experiments conducted at a pH of 6 and a temperature of 25 °C, and with a Cu/Cu_2_O/BC dosage of 100 mg, were used to study the impact of the initial dye concentration, catalyst addition, and temperature on the efficiency of the MO photocatalytic degradation. In addition, the impact of the initial concentration of the catalytic dye on the elimination of the MO was investigated. The impact of varying initial MO concentrations on the photocatalytic degradation of MO by Cu/Cu_2_O/BC is depicted in Figure 14a. The findings reveal that the optimal removal rate of 98.23% for MO is attained at 120 min when the initial concentration of MO is set at 50 mg/L. The maximal removal rate progressively drops as the initial MO concentration rises. Additionally, the maximum removal rate drops to 46.05% when the initial MO content rises to 200 mg/L. At the same time, the time required for the same removal rate increases. This is because the surface of Cu/Cu_2_O/BC is easily encapsulated by MO molecules in the high concentration of the MO solution, which reduces the contact surface of the Cu/Cu_2_O/BC catalyst with light and, thus, decreases the photocatalytic performance of Cu/Cu_2_O/BC.

The studies on the photocatalytic degradation of the MO were carried out at pH = 6, T = 25 °C, and with an initial MO concentration of 100 mg/L, to investigate the effect of the Cu/Cu_2_O/BC dosage of the catalyst on the elimination of the MO. Figure 14b exhibits the relationship between the Cu/Cu_2_O/BC dosage and the MO removal rate. When the Cu/Cu_2_O/BC dosage is 50 mg, the removal of MO reaches 69.78% after 300 min of photocatalysis. When the Cu/Cu_2_O/BC dosage is increased to 100 mg, the removal of MO reaches equilibrium, with the removal rate increasing significantly to 96.42%. However, when the catalyst Cu/Cu_2_O/BC dosage continues to increase, the MO removal rate begins to show a decreasing trend, which was deduced to be because the excess of the biochar-containing catalyst increases the turbidity of the solution, which then lowers the transmittance and affects the photocatalytic activity of the solution, causing the reduced degradation of the MO by the Cu/Cu_2_O/BC.

With pH = 6, an initial MO concentration of 100 mg/L, and a dose of 100 mg of Cu/Cu_2_O/BC, the impact of the Cu/Cu_2_O/BC on the photocatalytic process of the MO was examined at three different temperatures: 293.15 K, 313.15 K, and 333.15 K. The experimental findings are displayed in Figure 14c. The rate at which MO is removed by Cu/Cu_2_O/BC gradually increases from 95.16% to 97.32% as the temperature increases from 293.15 K to 333.15 K. This suggests that the photocatalytic process is heat-absorbing and that raising the temperature has a beneficial impact on MO removal. The reason was judged to be the increase in temperature activating molecules and accelerating the photocatalytic process.

**Figure 15:**
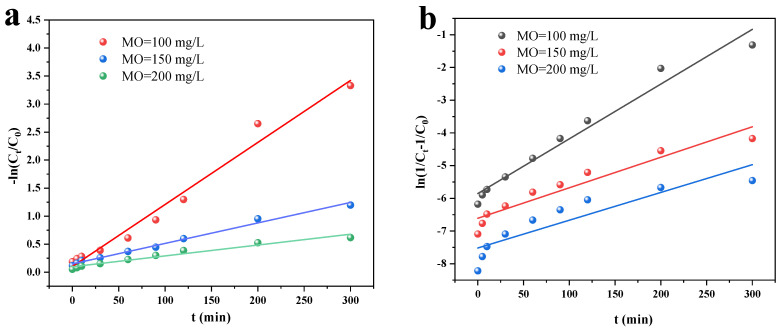
Photocatalytic kinetics of Cu/Cu_2_O/BC for MO removal by: (**a**) quasi-primary dynamics, and (**b**) quasi-secondary dynamics.

The photocatalytic kinetics of Cu/Cu_2_O/BC for MO removal were further investigated using the quasi-primary (Figure 15a) and quasi-secondary (Figure 15b) kinetic models with their linear expressions, as in Equations (13) and (14):

(13) $$-\ln(C_{t}/C_{0})=k_{1}t$$

(14) $$\ln(\frac{1}{C_{t}}-\frac{1}{C_{0}})=k_{2}t$$

where the slope $k_{1}$ and $k_{2}$ (1/min) are the rate constants for quasi-primary and quasi-secondary kinetic modelling, respectively, and *C*_0_ (mg/L) and *C_t_* (mg/L) are the initial concentration of MO and the concentration at time *t*.

Table 5 lists the model fit parameters. The quasi-primary kinetic model’s correlation coefficients are all greater than 0.98 and closer to 1, indicating that it is a superior match for explaining the photocatalytic process of MO elimination.

#### 3.2.4. MO Removal Mechanisms

**Figure 16:**
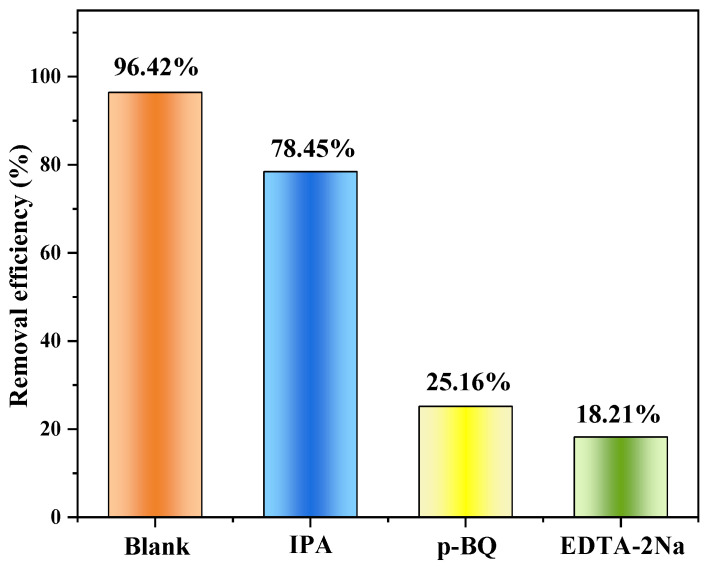
The active substances in Cu/Cu_2_O/BC quenched by IPA, p-BQ, and EDTA-2Na.

Capture experiments in which traps are added to capture reactive substances are widely used to study catalytic mechanisms. It has been shown that pollutants can be degraded by various active substances, such as hydroxyl radicals (•OH), holes (h^+^), and superoxide radicals (•O_2_^−^), through oxidation and/or reduction reactions. In order to reveal the role of reactive substances in the degradation of MO by Cu/Cu_2_O/BC, trapping experiments were performed by using isopropanol (IPA, 1 mmol) as a hydroxyl radical (•OH) scavenger, p-benzoquinone (p-BQ, 1 mmol) as a scavenger of superoxide radicals (•O_2_^−^) and ethylenediaminetetraacetic acid (EDTA-2Na, 1 mmol) as a hole (h^+^) scavenger. The capture experiment results are displayed in Figure 16, where it is evident that the presence of IPA has a small impact on MO degradation, indicating that MO degradation by •OH is minimal. The degradation efficiency of MO decreases from 96.42% (without additions) to 25.16% and 18.21%, respectively, after 5 h in the presence of the EDTA-2Na and p-BQ environment, suggesting that h^+^ and •O_2_^−^ are important players in the photocatalytic degradation process.

**Figure 17:**
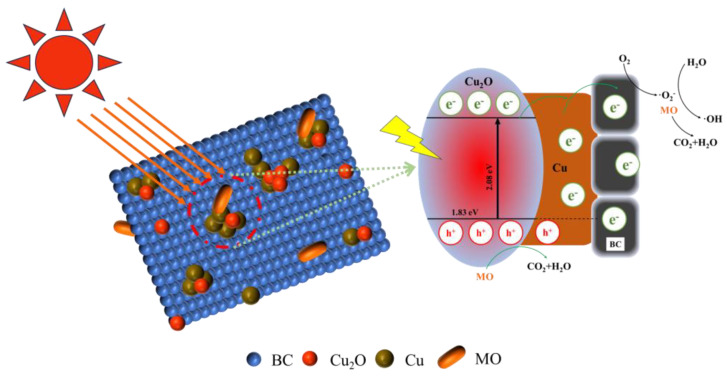
Removal mechanism of Cu/Cu_2_O/BC on photocatalytic degradation of MO.

A potential synergistic adsorption–photocatalytic degradation pathway is suggested based on the aforementioned investigations, and it is depicted in Figure 17. Rice husk biochar, as an excellent carrier, provides more channels for Cu/Cu_2_O to adsorb MO [45]. Multiple MO adsorption active sites are present in Cu/Cu_2_O/BC due to complexation, ion exchange, and electrostatic attraction, which enables MO molecules to fully make contact with Cu/Cu_2_O/BC. On the other hand, Cu/Cu_2_O/BC quickly produces photogenerated electrons when exposed to visible light. While holes (h^+^) react with H_2_O or OH^−^ adsorbed on the surface of Cu/Cu_2_O/BC to make hydroxyl radicals (•OH), generated electrons (e^−^) convert O_2_ to superoxide radicals (•O_2_^−^). To eliminate the MO organic pollutants, the strong oxidizing active ingredient of radical •OH breaks down the MO chromophores into tiny, non-toxic molecules [46]. The reaction equations are shown in Equations (15)–(21), as follows:

(15) $${\mathrm{Cu}}_{2}O+\mathrm{hv}\to h^{+}({\mathrm{Cu}}_{2}O)+e^{-}({\mathrm{Cu}}_{2}O)$$

(16) $$h^{+}({\mathrm{Cu}}_{2}O)+\mathrm{Cu}+\mathrm{BC}\to e^{-}(\mathrm{Cu})+e^{-}(\mathrm{BC})+{\mathrm{Cu}}_{2}O$$

(17) $$e^{-}(\mathrm{Cu})+O_{2}\to \mathrm{Cu}+\cdot O_{2}^{-}$$

(18) $$e^{-}(\mathrm{BC})+O_{2}\to \mathrm{BC}+\cdot O_{2}^{-}$$

(19) $$\cdot O_{2}^{-}+H_{2}O\to \cdot \mathrm{OH}+{\mathrm{OH}}^{-}$$

(20) $$\mathrm{MO}+h^{+}({\mathrm{Cu}}_{2}O)+\cdot O_{2}^{-}+\cdot \mathrm{OH}\to {\mathrm{CO}}_{2}+H_{2}O$$

(21) $$h^{+}({\mathrm{Cu}}_{2}O)+\mathrm{Cu}\to h^{+}(\mathrm{Cu})+{\mathrm{Cu}}_{2}O$$

### 3.3. Reusability and Stability

**Figure 18:**
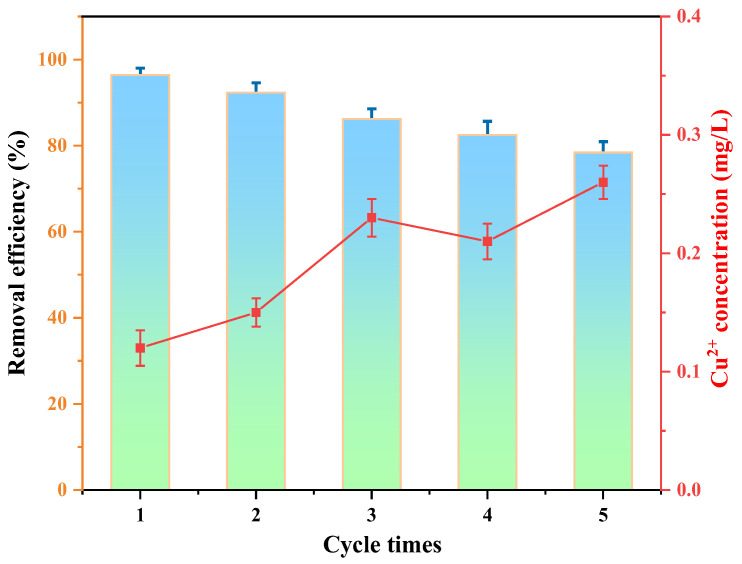
Relationships in the recycling degradation of MO by Cu/Cu_2_O/BC.

In order to investigate the reusability and stability of the Cu/Cu_2_O/BC, after a Cu/Cu_2_O/BC adsorption–photocatalytic degradation of 100 mg/L MO, the photodegraded solution was collected, the catalyst was recovered by the extraction filtration method and washed, dried and reused, and the MO removal experiment was carried out through five consecutive rounds of adsorption–photocatalytic degradation. Figure 18 illustrates the correlation between the MO removal rate and the recycling of Cu/Cu_2_O/BC. An overall MO removal rate of 96.42% was achieved in the first run of the removal experiment, in 6 h. In the fifth run, the MO removal efficiency still reached 78.41%, showing the excellent reusability of the Cu/Cu_2_O/BC. This can be explained by two factors: first, during the photocatalytic process, the adsorbed MO was completely mineralized, exposing the adsorption sites once more; second, the Cu/Cu_2_O/BC exhibited excellent photo-corrosion resistance, guaranteeing that its heterojunction interface maintained high photo-reactivity throughout the cycling process.

After each reaction cycle, we monitored the concentration of leached copper ions in the solution. Following the initial cycle, it was clear that the concentration of leached copper ions measured 0.12 mg/L. Furthermore, it was kept at 0.26 mg/L after the fifth cycle, which is in full compliance with the provisions of China’s “Groundwater Quality Standard” (GB/T 14848-2017 [47]) on the content of copper in groundwater, indicating that the catalyst has good stability and recyclability.

## 4. Conclusions

The Cu/Cu_2_O/BC nanocomposites were successfully prepared through the impregnation method by immersing rice husk biochar in a copper salt solution and using Na_2_S_2_O_4_ as a single reducing agent to generate Cu/Cu_2_O nanoparticles in situ on the biochar, before using the nanocomposites as adsorption photocatalysts for the removal of organic dyes in aqueous solutions, such as MO. Various characterization approaches were used to investigate the effects of temperature, pH, and initial concentration on the adsorption–photocatalytic process. Additionally, the removal mechanism was analyzed by using the reaction kinetics, adsorption isotherm, and thermodynamics to study the adsorption process and the photocatalytic process. The following conclusions were drawn:

(1) At a mass ratio of BC to Cu/Cu_2_O of 0.2, the synthesized Cu/Cu_2_O/BC photocatalyst exhibits the highest adsorption–photocatalytic action. At pH = 6 and T = 25 °C, 100 mg of Cu/Cu_2_O/BC can remove 95% of the MO and 88.26% of the COD from the aqueous solution at an initial concentration of 100 mg/L.
(2) The maximal removal rate in the dark adsorption method reduces slightly as the temperature rises. The removal rate rises with increasing temperature in the adsorption–photocatalysis synergistic treatment of MO, indicating that the adsorption is an exothermic reaction and that the photocatalysis is an adsorptive one, with the photocatalysis process being more affected than the adsorption process. The greatest removal effect occurs at pH = 3.0, while the adsorption of MO by Cu/Cu_2_O/BC is inhibited when the pH is greater than 7. The MO removal rate in the adsorption process rises when decreasing the pH of the solution.
(3) The quasi-primary kinetic model and the quasi-secondary kinetic model both suitably describe the adsorption and photocatalytic processes, respectively. Additionally, the adsorption isotherm more closely resembles the Langmuir model, indicating that MO is mostly chemisorbable by Cu/Cu_2_O/BC. The Langmuir model determines the *q*_max_ of MO to be 45.45 mg/g. According to the thermodynamic analysis, entropy, not enthalpy, is primarily responsible for controlling the spontaneous adsorption process during removal.
(4) When MO is adsorbed on Cu/Cu_2_O/BC, and when Cu_2_O is exposed to light radiation, photogenerated electrons move to produce holes, with the electrons transferred to Cu and biochar to react with O_2_ to produce the active substance of •O_2_^−^, and MO is then oxidized and reduced by h^+^ and •O_2_^−^ to H_2_O and CO_2_. A high MO removal rate of 78.41% can remain after five cycles of treatment, indicating the excellent recyclability of the Cu/Cu_2_O/BC.

## Figures and Tables

**Table 1:** Kinetics parameters of Cu/Cu_2_O/BC in adsorption of MO.

*C*_0_	Quasi-First-Order Kinetics Model			Quasi-Second-Order Kinetics Model		
	*q*_1_	*k*_1_	*R* ^2^	*q*_2_	*k*_2_	*R* ^2^
50	39.201	0.00338	0.986	37.74	0.00215	0.990
100	41.134	0.00292	0.983	45.93	0.00182	0.996
150	51.516	0.00239	0.977	54.88	0.00158	0.998

Note: *C*_0_ is in mg/L, *q*_1_ and *q*_2_ are in mg/g, *k*_1_ is in 1/min, and *k*_2_ is in mg/g·min.

**Table 2:** Effects of relevant parameters of Cu/Cu_2_O/BC on MO adsorption in the internal diffusion phase.

*C*_0_	Internal Diffusion Model					
	*k*_d1_	*I*_1_	*R* ^2^	k_d2_	I_2_	*R* ^2^
50	2.062	−2.153	0.979	0.481	25.525	0.993
100	2.160	0.555	0.983	0.286	35.126	0.950
150	2.351	−0.594	0.990	0.572	31.963	0.993

Note: *C*_0_ is in mg/L, and *k*_d1_ and *k*_d2_ are in mg/g·min^0.5^.

**Table 3:** Fitting parameters of the Langmuir and Freundlich models.

Langmuir Model Constant				Freundlich Model Constant		
*q*_max_	*k*_L_	*r* ^2^	*R*_L_	1/*n*	*k*_F_	*r* ^2^
45.454	0.5095	0.996	0.01–0.455	0.108	27.855	0.853

**Table 4:** Thermodynamic fitting results of MO adsorption by Cu/Cu_2_O/BC.

*T* (K)	*Kc*	Δ*G*^0^ (kJ/mol)	Δ*S*^0^ (J/mol·K)	Δ*H*^0^ (kJ/mol)
293.15	2.926	−2.616	−71.733	−23.578
303.15	1.955	−1.689		
313.15	1.578	−1.187		

**Table 5:** Kinetic parameters of Cu/Cu_2_O/BC photocatalysis for MO removal.

*C*_0_ (mg/L)	Quasi-Primary Kinetic Model		Quasi-Secondary Kinetic Model	
	*k*_1_ (min^−1^)	*R* ^2^	*k*_2_ (min^−1^)	*R* ^2^
100	1.11 × 10^−2^	0.980	1.67 × 10^−2^	0.972
150	3.66 × 10^−3^	0.991	9.32 × 10^−3^	0.921
200	1.94 × 10^−3^	0.962	8.49 × 10^−3^	0.789

## Data Availability

The original contributions presented in the study are included in the article, further inquiries can be directed to the corresponding author.
